# Awareness of voluntary action, rather than body ownership, improves motor control

**DOI:** 10.1038/s41598-020-79910-x

**Published:** 2021-01-11

**Authors:** Kazumichi Matsumiya

**Affiliations:** 1 Graduate School of Information Sciences, Tohoku University, 6-3-09 Aoba, Aramaki-aza, Aoba-ku, Sendai, 980-8579 Japan; 2 Japan Science and Technology Agency, Precursory Research for Embryonic Science and Technology (PRESTO), 6-3-09 Aoba, Aramaki-aza, Aoba-ku, Sendai, 980-8579 Japan

**Keywords:** Human behaviour, Cognitive neuroscience, Motor control

## Abstract

Awareness of the body is essential for accurate motor control. However, how this awareness influences motor control is poorly understood. The awareness of the body includes awareness of visible body parts as one’s own (sense of body ownership) and awareness of voluntary actions over that visible body part (sense of agency). Here, I show that sense of agency over a visible hand improves the initiation of movement, regardless of sense of body ownership. The present study combined the moving rubber hand illusion, which allows experimental manipulation of agency and body ownership, and the finger-tracking paradigm, which allows behavioral quantification of motor control by the ability to coordinate eye with hand movements. This eye–hand coordination requires awareness of the hand to track the hand with the eye. I found that eye–hand coordination is improved when participants experience a sense of agency over a tracked artificial hand, regardless of their sense of body ownership. This improvement was selective for the initiation, but not maintenance, of eye–hand coordination. These results reveal that the prospective experience of explicit sense of agency improves motor control, suggesting that artificial manipulation of prospective agency may be beneficial to rehabilitation and sports training techniques.

## Introduction

Knowing our body’s state is fundamental to motor control^1^. Without knowledge of our body’s state, we would be unable to control our body parts accurately. To estimate the body’s state for motor control, the brain must distinguish between signals generated as a consequence of voluntary body part movement and signals arising from events in the external world. For this differentiation, two brain mechanisms can be considered. First, when we actively move our body part, the brain predicts the sensory consequences of the body movements through our intentions (or motor commands)^1,2^. Comparison between motor commands and sensory consequences of the body movements leads to the awareness that we are the author of the body movements (sense of agency)^3^. Second, the integration of signals from different sensory modalities (e.g., vision, touch, and position sense [proprioception]) originating from the same body part leads to the awareness that the body part is part of one’s own body (sense of body ownership)^4–7^. However, how the senses of agency and body ownership contribute to knowing the body’s state for motor control remains unclear.

Sense of agency has been explained by using current computational models of motor control, such as the comparator model^3^. According to these models, sense of agency is generally thought to be generated when voluntary actions match outcomes in all aspects of motor control including movement initiation and ongoing movement^8,9^. However, recent evidence has suggested that sense of agency may be linked to initiation of voluntary action^3,10^. During movement initiation, the frontal and parietal areas of the brain play a critical role in motor control^2^. Activity in these areas has been strongly correlated with subjective intention to move before movement execution^11,12^. However, what aspects of motor action involve sense of agency over body parts remains unclear. Although a more recent fMRI and rTMS study addressed this issue with implicit sense of agency^13^, no study investigates the relationship with explicit sense of agency.

Moreover, it remains unclear whether sense of body ownership affects motor control. Indeed, evidence is mixed concerning the contribution of this sense to estimating the body’s state for motor control. In recent years, there has been growing evidence that motor control is affected by sense of body ownership from several modalities. Sense of ownership of a visible hand affects endpoint errors^14,15^ and initial direction^16^ of reaching movements. The distance between the thumb and index finger during grasping movements is modulated by sense of body ownership^17^. Attenuation of self-generated tactile sensations depends on sense of body ownership^18–21^. Moreover, there is neuropsychological evidence supporting the view that body ownership plays a key role in motor consciousness^22^. For example, a monothematic delusion of body ownership affects motor control^22^. All these findings are consistent with the role of body ownership in motor control^23^. However, patients with motor paralysis preserve their sense of body ownership^24,25^, although other studies suggest the opposite view in the cases of patients with spinal cord injury^26^ and hemiplegia^27^. In healthy participants, motor control is adaptive^28^ and online control of motor actions^29,30^ is possible while keeping the participant’s own hand invisible to the participant. In these cases, estimation of the body’s state for motor control does not seem to require the visual component of body ownership. There is also another clinical evidence for the behavioral dissociation between somatoparaphrenia (disturbed sense of body ownership) and anosognosia for hemiplegia (disturbed sense of agency)^31^. Furthermore, other recent studies did not find effects of body ownership on reaching^32^ or pointing^33^ behavior. Hence, these previous studies have not reached consensus on whether body ownership affects the motor system. Behavioral tasks in these studies all involved voluntary actions. Such voluntary actions typically generate sense of agency over a participant’s own hand, and this sense of agency crucially influences the motor system^23,34,35^. This makes it difficult to ascertain whether body ownership truly affects the motor system.

Here, I take a different approach to determine the functional roles of agency and body ownership in motor control. Consider participants tracking a finger of a moving artificial hand with their eyes while the artificial hand moves together with the participant’s real hidden hand. This situation induces both eye–hand coordination and the illusory experiences of agency and ownership over the artificial hand [the moving rubber hand illusion^36^ (RHI)].

Eye–hand coordination allows behavioral quantification of motor control for various aspects of motor control, including movement initiation and ongoing movement. When participants track their finger with their eyes, anticipatory smooth pursuit eye movements are generated for stabilizing the image of the tracking finger on the fovea. The eye tracking of self-generated hand movements has been shown to be better than the tracking of external movements^37–39^. This indicates that the eye movement system uses information from hand motor commands in eye–hand coordination. The benefit includes reduced eye latency, increased pursuit gain (eye velocity/hand velocity), and decreased number of saccadic eye movements. The enhanced performance in eye tracking of self-generated hand movements can be used to assess the quality of hand motor control. Moreover, eye latency is related to the initiation of eye–hand coordination. Pursuit gain and the number of saccades are related to the maintenance of eye–hand coordination. Thus, eye–hand coordination can also be used to assess the quality of motor control for movement initiation and ongoing movement.

The moving RHI allows experimental manipulation of agency and body ownership. When the artificial hand moves synchronously with the real hidden hand, illusory ownership of the artificial hand is induced. Importantly, this illusory ownership of the artificial hand can occur even without a sense of agency over the artificial hand^36,40^. A recent study has also used a similar manner to manipulate sense of body ownership alone, while maintaining sense of agency^41^. Sense of agency over the artificial hand can also occur even without a sense of body ownership over the artificial hand^36,40^. The moving RHI enables examination for a pure effect of body ownership or agency on motor control.

## Results

### Experiment 1

#### Methods

**Figure 1:**
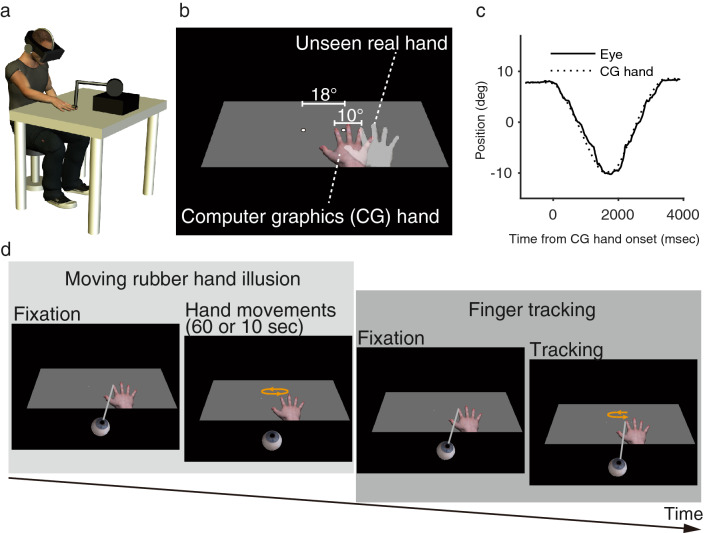
Apparatus, stimuli, and procedure. (**a**) Participants binocularly viewed the visual stimulus presented through a head-mounted display and placed their right hand on a table. Their right index finger was attached to the arm of a force-feedback device. (**b**) A computer graphics (CG) hand and two white points were presented on a gray surface in the virtual environment. The right index finger of the CG hand was placed 10° to the left of the participant’s unseen right index finger. One white point served as the starting point of eye and hand movements, and the other served as the turning point. (**c**) Sample result of eye and hand positions for one participant. (**d**) On a given trial, the participant’s hand was moved actively or passively to induce the moving rubber hand illusion. Then, the participants were instructed to track the CG hand movements with pursuit eye movements.

Participants were shown a realistic life-sized computer graphics (CG) hand through a head-mounted display (HMD; Fig. 1a). The CG hand was placed 10° to the left of the participant’s unseen right hand (Fig. 1b) and was configured similarly to the participant’s hand. Participants were instructed to track the index finger of the CG hand with their eyes while their hidden hand was moved (Fig. 1c and Supplementary Movie 1). Variations were made to the mode of the participant’s hand movement (active or passive) and the temporal congruence between the movements of the participant’s hand and the CG hand (synchronous or asynchronous). In the active condition, participants controlled the movements of the CG hand by moving their hidden hand. In the passive condition, the participant’s entire right arm, which was hidden, was moved using the arm of a force-feedback device attached to the participant’s right index finger. In the synchronous condition, the CG hand was moved synchronously with the participant’s hand movements. In the asynchronous condition, the participant’s hand was moved actively or passively, but the movements of the CG hand were temporally delayed by 0.5 s. Thus, four conditions were defined in total: active-synchronous, active-asynchronous, passive-synchronous, and passive-asynchronous.

In experiment 1, to manipulate the senses of body ownership and agency for the CG hand before tracking the finger of the CG hand with the participant’s eyes, participants first repeatedly moved their right hand from side to side for 60 s for the first trial (for 10 s after the first trial) while they observed the CG hand moving in synchrony or asynchrony with the movements of their hand (Fig. 1d). After each moving RHI trial, the participant’s right hand started to move to either the left or the right and back to the starting point, and the participants were instructed to track the tip of the index finger of the CG hand by smooth pursuit eye movement (Fig. 1d). After all the finger-tracking trials, the participants answered questionnaire items to rate subjective aspects of the CG hand (Table 1). For both cases of ownership and agency, the two questionnaire items were relevant to the illusory experience (illusion questions) and the remaining three served as controls (control questions). The ownership or agency index was calculated by subtracting the median score of responses to the control questions from the median score of responses to the illusion questions in a similar way to previous studies^18,36^. Taking into account the participants’ responses to these control items reduces any effect of suggestibility or compliance and thus gives a more reliable estimate of the strength of ownership or agency. If senses of body ownership and agency affect motor control, all aspects of eye–hand coordination should be improved by these senses.

**Table 1:** Questionnaire items for measuring subjective aspects of the computer graphics (CG) hand in experiment 1.

Category	Questionnaire items
Ownership	I felt that the CG hand was my hand
	The felt position of the real hand was located on the CG hand
Ownership control	The real hand was drifting toward the CG hand
	The real hand was turning into a computer graphics
	It seemed that I had more than one right hand
Agency	The CG hand moved as if it was obeying my will
	I felt as if I was controlling the movements of the CG hand
Agency control	I felt as if the CG hand was controlling my will
	I could sense the movement from somewhere between my real hand and the CG hand
	It seemed as if the CG hand had a will of its own

Participants rated their level of agreement with each item on a 7-point scale, ranging from -3 (strongly disagree) to + 3 (strongly agree).

#### Results

**Figure 2:**
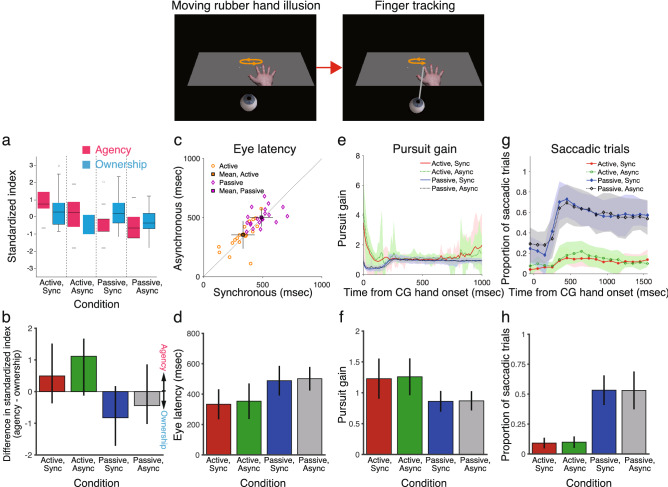
Results of experiment 1. (**a**, **b**) Strength of the rated ownership and agency of the computer graphics (CG) hand shown as box-and-whisker plots for the four conditions. The ownership and agency indexes were defined as the difference in ratings between the illusion and control questions. (**a)** The standard score (z-score) was calculated for each of the ownership (blue) and agency (pink) indexes, which were defined as the standardized index. Horizontal black bars represent medians, and the boxes denote the interquartile ranges. Whiskers extend to the farthest data points within 1.5 times the interquartile range from the top and bottom edges of the boxes. Individual open circles represent outliers. (**b)** The standardized ownership index was subtracted from the standardized agency index. Colored bars represent the medians, and the error bars represent the interquartile ranges. (**c**, **d**) Eye latency, as defined by eye onset time relative to CG hand onset time. Orange and purple symbols represent the active and passive hand movement conditions, respectively, for individual participants. Square symbols represent the mean ± standard deviation. (**e**, **f**) Pursuit gain, defined as eye velocity divided by CG hand velocity after excluding saccades in velocity traces. (**e**) Shaded areas represent standard deviation. (**f**) Shows the average pursuit gain in the range of 0 to 1000 ms. (**g**, **h**) Proportion of saccadic trials in the range of − 200 to 1600 ms from CG hand onset. Results are the mean ± standard deviation in Figs. 2d–h (*n* = 20).

For ownership rating, analysis of the questionnaire data revealed a significant main effect of timing condition (Friedman test, χ^2^ = 10.76, *df* = 1, *n* = 20, *P* < 0.01), but there was no significant main effect of action condition (χ^2^ = 0.07, *df* = 1, *n* = 20, *P* = 0.80 *n.s.*) (Fig. 2a; see Supplementary Figs. 1 and 2 for further details). There was no significant interaction between action and timing conditions (Regression analysis, *t*_*19*_ = 0.79, *P* = 0.44 *n.s.*). In the active-synchronous and passive-synchronous conditions, each median of the ownership rating was below + 1, which suggests that on average, participants tended to disagree with the statements that the illusion occurred. This is because of large variability on measures of the RHI across individuals^42–46^. For agency rating, the analysis revealed a significant main effect of action condition (χ^2^ = 22.86, *df* = 1, *n* = 20, *P* < 0.001), but there was no significant main effect of timing condition (χ^2^ = 3.32, *df* = 1, *n* = 20, *P* = 0.069 *n.s.*). There was no significant interaction between action and timing conditions (*t*_*19*_ = 1.39, *P* = 0.17 *n.s.*). These results indicate that (i) sense of body ownership over the CG hand is sensitive to timing but not action, and (ii) sense of agency over the CG hand is sensitive to action but not timing, suggesting that the moving RHI technique used in the present study can manipulate senses of body ownership and agency independently. These results are consistent with previous findings^36,40,47^. They also show that the participants’ agency index in the synchronous condition did not significantly differ from that in the asynchronous condition, although previous studies that used asynchrony to manipulate sense of agency^19,48^. This is not to say that asynchrony could not be used to manipulate sense of agency, but the present results suggest that asynchrony was not effective in preventing induction of sense of agency. Indeed, there was a marginally significant difference in agency index between the synchronous and asynchronous conditions (χ^2^ = 3.32, *df* = 1, *n* = 20, *P* = 0.069), if another statistical criterion was applied to the data.

To directly compare the agency index to the ownership index, I calculated the standard score (z-score) for each of the ownership and agency indexes and then subtracted the standardized ownership index from the standardized agency index. Analysis of the median difference in standardized indexes between agency and ownership revealed that the difference in standardized indexes was significantly larger in the active-asynchronous condition than in the passive-synchronous condition (Fig. 2b; Bonferroni corrected median test: *z* = 8.12, *P* < 0.05), suggesting that the agency index is larger than the ownership index in the active-asynchronous condition and that the ownership index is larger than the agency index in the passive-synchronous condition. Of particular note, (1) participants experienced senses of agency and ownership for the CG hand in the active-synchronous condition, (2) participants experienced a sense of agency, but not a sense of ownership, for the CG hand in the active-asynchronous condition, (3) participants experienced a sense of ownership, but not a sense of agency, for the CG hand in the passive-synchronous condition, and (4) participants experienced neither senses of agency nor ownership for the CG hand in the passive-asynchronous condition (constituting a control).

I analyzed how well participants coordinated their eyes with the CG hand movements (see Methods for further details). First, the temporal predictability of the CG hand movements was assessed by analyzing eye latencies, which were defined by eye onset time relative to CG hand onset time (Fig. 2c,d and Supplementary Fig. 3). Analysis of eye latency revealed a significant main effect of action (active vs passive) condition (Friedman test, *χ*^2^ = 31.84, *df* = 1, *n* = 20, *P* < 0.001), but no significant main effect of timing (synchronous vs asynchronous) condition (*χ*^2^ = 0.65, *df* = 1, *n* = 20, *P* = 0.42 *n.s.*). There was no significant interaction between action and timing conditions (Regression analysis, *t*_*19*_ = -0.17, *P* = 0.86 *n.s.*). Eye latency in the active-asynchronous condition was comparable to that in the active-synchronous condition but was significantly shorter than that in the passive-asynchronous condition (Bonferroni corrected paired Dunn test: *z* = –4.16, *P* < 0.001). Eye latency in the passive-synchronous condition was comparable to that in the passive-asynchronous condition but was significantly longer than in the active-synchronous and active-asynchronous conditions (*z* = –5.27, *P* < 0.001 and *z* = –3.80, *P* < 0.01, respectively). The finding that eye latency is almost the same in the active-asynchronous and active-synchronous conditions is consistent with the results of previous studies^39,49^, suggesting that pursuit eye movements are mostly under visual control, although eye movements are affected by self-generated hand movements. These results demonstrate that eye latency is shorter under the active-asynchronous condition, but not under the passive-synchronous condition.

Second, pursuit gain (eye velocities/CG hand velocities) was analyzed to assess the quality of predictive pursuit eye movements (Fig. 2e,f). Because saccadic eye movements also contribute to catching up with the position of the CG hand during pursuit eye movements, it is difficult to assess the spatial predictability of CG hand movement for pursuit eye movements using position measurement alone. To address this problem, pursuit gain was analyzed after excluding saccades. Analysis of pursuit gain revealed a significant main effect of action condition (*χ*^2^ = 34.93, *df* = 1, *n* = 20, *P* < 0.001), but no significant main effect of timing condition (*χ*^2^ = 0.31, *df* = 1, *n* = 20, *P* = 0.58 *n.s.*). There was no significant interaction between action and timing conditions (*t*_*19*_ = -0.41, *P* = 0.68 *n.s.*). Pursuit gain in the active-asynchronous condition was comparable to that in the active-synchronous condition but was significantly different than that in the passive-asynchronous conditions (*z* = 5.39, *P* < 0.001). Pursuit gain in the passive-synchronous condition was comparable to that in the passive-asynchronous condition but was significantly different than in the active-synchronous and active-asynchronous conditions (*z* = 3.92, *P* < 0.01 and *z* = 4.90, *P* < 0.001, respectively). These results indicate that pursuit gains are larger in the active-asynchronous condition, but not in the passive-synchronous condition.

Finally, I analyzed the number of saccadic eye movements, the rate of which is another index of smooth pursuit performance. Previous studies have shown a low number of saccades for successful eye-tracking of self-generated hand movements^37,38,50–52^. To calculate the proportion of saccadic trials, I counted the number of trials that had at least one saccade and divided this by the total number of trials. Figure 2g,h show the proportion of saccadic trials across the four conditions. Analysis of the proportion of saccadic trials revealed a significant main effect of action condition (*χ*^2^ = 58.24, *df* = 1, *n* = 20, *P* < 0.001), but no significant main effect of timing condition (*χ*^2^ = 0.77, *df* = 1, *n* = 20, *P* = 0.38 *n.s.*). There was no significant interaction between action and timing conditions (*t*_*19*_ = -0.24, *P* = 0.81 *n.s.*). Proportion of saccadic trials in the active-asynchronous condition was comparable to that in the active-synchronous condition but was significantly different than that in the passive-asynchronous conditions (*z* = –4.53, *P* < 0.001). Proportion of saccadic trials in the passive-synchronous condition was comparable to that in the passive-asynchronous condition but was significantly different than in the active-synchronous and active-asynchronous conditions (*z* = –5.27, *P* < 0.001 and *z* = –4.29, *P* < 0.001, respectively). These results indicate that there were fewer saccades in the active-asynchronous condition than in the passive-synchronous condition.

**Figure 3:**
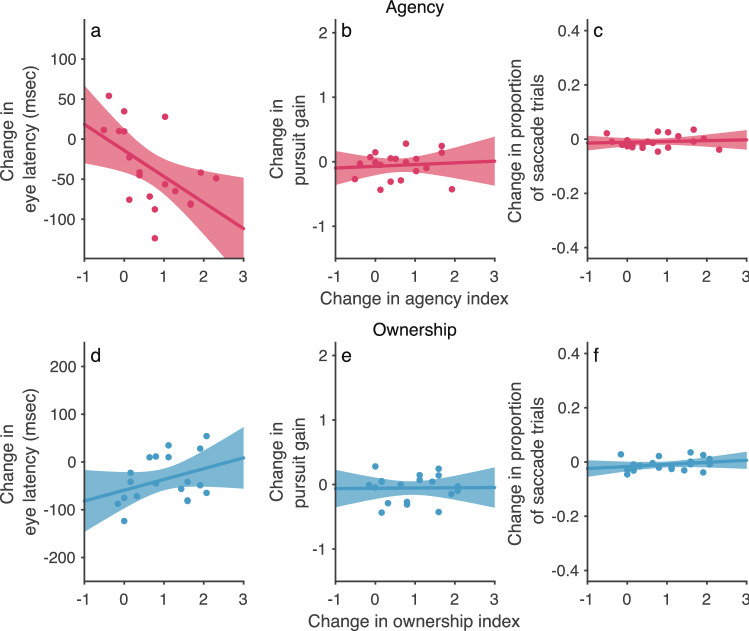
Correlation between body awareness and eye–hand coordination performance in experiment 1 (*n* = 20). (**a**–**c**) Agency. (**d**–**f**) Ownership. Change in eye–hand coordination performance [i.e., eye latency (**a**, **d**), pursuit gain (**b**, **e**), and proportion of saccadic trials (**c**, **f**)] was defined as the change in performance when the sense of agency or the sense of body ownership became stronger in the active-synchronous condition compared with the active-asynchronous condition. One participant was excluded from Spearman’s rank correlation analysis for both eye latency and pursuit gain (see Methods for details).

The above results show that eye–hand coordination performance is improved in the active-asynchronous condition, in which participants experienced a sense of agency over the CG hand. However, it is unclear whether active movement or sense of agency improved eye–hand coordination performance. To address this issue, I analyzed the correlation between strength of sense of agency and eye–hand coordination performance. In this analysis, I examined whether the eye–hand coordination performance improves when sense of agency increases in the active-synchronous condition compared with the active-asynchronous condition. Surprisingly, eye latency was significantly correlated with sense of agency (Fig. 3a: Spearman correlation test, *r*_*s*_ = -0.58, *N* = 19, *P* < 0.01), but pursuit gain and proportion of saccadic trials were not (Fig. 3b,c: *r*_*s*_ = 0.069, *N* = 19, *P* = 0.78 *n.s.* for pursuit gain and *r*_*s*_ = 0.002, *N* = 20, *P* = 0.995 *n.s.* for proportion of saccadic trials). These results suggest that (i) eye latency is affected by sense of agency, and (ii) pursuit gain and proportion of saccadic trials are affected by active movement, not sense of agency. Also, there were no significant correlations between sense of ownership and eye–hand coordination for eye latency (*r*_*s*_ = 0.33, *N* = 19, *P* = 0.16 *n.s.*), pursuit gain(*r*_*s*_ = 0.12, *N* = 19, *P* = 0.63 *n.s.*), and the proportion of saccadic trials (*r*_*s*_ = 0.25, *N* = 20, *P* = 0.29 *n.s.*) (Fig. 3d–f). Eye latency is related to the initiation of eye–hand coordination. In contrast, pursuit gain and the proportion of saccadic trials are related to the maintenance of eye–hand coordination. The difference in movement distance between the active and passive conditions was not correlated with sense of agency or with sense of body ownership (see Supplementary Fig. 8 for details). Thus, these findings suggest that sense of agency selectively contributes to the initiation of eye–hand coordination.

It may be of concern that improved pursuit eye movement performance in the active-asynchronous condition was generated by adaptation in a positional offset between the hidden hand and the CG hand, but not by sense of agency over the CG hand. In the active-asynchronous condition, to induce a sense of agency over the CG hand, participants repeatedly moved their hand in a horizontal direction asynchronously with the movements of the CG hand before eye-tracking of the index finger of the CG hand. This leads to adaptation in perceived hand position, that is, the perceived location of the real hand is displaced toward the CG hand. After the adaptation, eye-tracking was tested in the same horizontal direction as the adaptation. Therefore, good eye-tracking in the active-asynchronous condition might be caused by adapting the position of the hand. To rule out this possibility, I conducted an additional experiment.

### Experiment 2

#### Methods

For experiment 2, the design involved adapting the position of the hand in the vertical spatial direction using the RHI procedure, and then testing eye-tracking in the horizontal spatial direction (see Methods and Supplementary Movie 2 for details). This distinguished spatial adaptation produced by the RHI procedure from temporal factors that induce the RHI. If eye-tracking performance were improved by sense of agency over the CG hand or active movement rather than by adaptation to the hand position, I would expect eye-tracking performance to be still as good in the active-asynchronous condition as in the active-synchronous condition. As expected, eye latency, pursuit gain, and the number of saccades in the active-asynchronous condition were comparable to those in the active-synchronous condition.

#### Results

**Figure 4:**
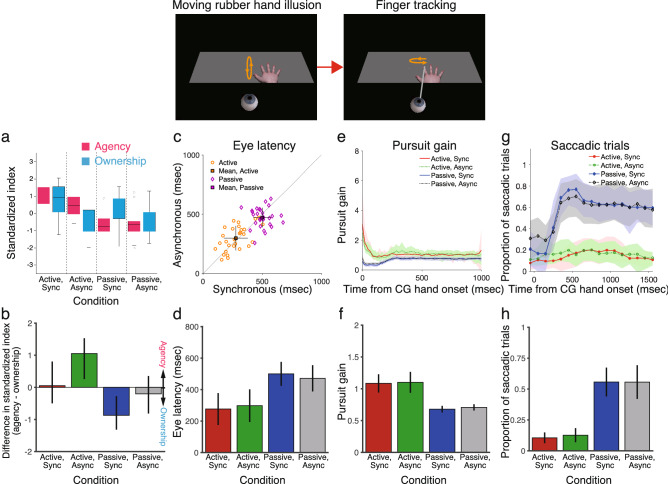
Results of experiment 2. (**a**, **b**) Strength of the rated ownership and agency of the computer graphics (CG) hand shown as box-and-whisker plots for the four conditions. The ownership and agency indexes were defined as the difference in ratings between the illusion and control questions. (**a**) The standard score (z-score) was calculated for each of the ownership (blue) and agency (pink) indexes, which were defined as the standardized index. Horizontal black bars represent medians, and the boxes denote the interquartile ranges. Whiskers extend to the farthest data points within 1.5 times the interquartile range from the top and bottom edges of the boxes. Individual open circles represent outliers. (**b**) The standardized ownership index was subtracted from the standardized agency index. Colored bars represent the medians, and the error bars represent the interquartile ranges. (**c**, **d**) Eye latency, as defined by eye onset time relative to CG hand onset time. Orange and purple symbols represent the active and passive hand movement conditions, respectively, for individual participants. Square symbols represent the mean ± standard deviation. (**e**, **f**) Pursuit gain, defined as eye velocity divided by CG hand velocity after excluding saccades in velocity traces. (**e**) Shaded areas represent standard deviation. (**f**) Shows the average pursuit gain in the range of 0 to 1000 ms. (**g**, **h**) Proportion of saccadic trials in the range of − 200 to 1600 ms from CG hand onset. Results are the mean ± standard deviation in (**d**–**h**) (*n* = 29).

Participants answered questionnaire items to rate subjective aspects of the CG hand in experiment 2 in a similar manner to that of experiment 1, but with more questionnaire items (Table 2). The results of the questionnaire data in experiment 2 were consistent with those of experiment 1 (Fig. 4a,b; see Supplementary Figs. 4–5 for details).

**Table 2:** Questionnaire items for measuring subjective aspects of the computer graphics (CG) hand in experiment 2.

Category	Questionnaire items
Ownership	I felt that the CG hand was my hand
	The felt position of the real hand was located on the CG hand
	I felt as if I was looking at my own hand
	I felt as if the CG hand was part of my body
Ownership control	The real hand was drifting toward the CG hand
	The real hand was turning into a computer graphics
	It seemed that I had more than one right hand
	I felt as if my right hand had disappeared
Agency	The CG hand moved as if it was obeying my will
	I felt as if I was controlling the movements of the CG hand
	I felt as if I was causing the CG hand movement I saw
	I expected the CG hand to move whenever I moved my hand
Agency control	I felt as if the CG hand was controlling my will
	I could sense the movement from somewhere between my real hand and the CG hand
	It seemed as if the CG hand had a will of its own
	It seemed as if the CG hand was controlling my movements

Participants rated their level of agreement with each item on a 7-point scale, ranging from -3 (strongly disagree) to + 3 (strongly agree).

**Figure 5:**
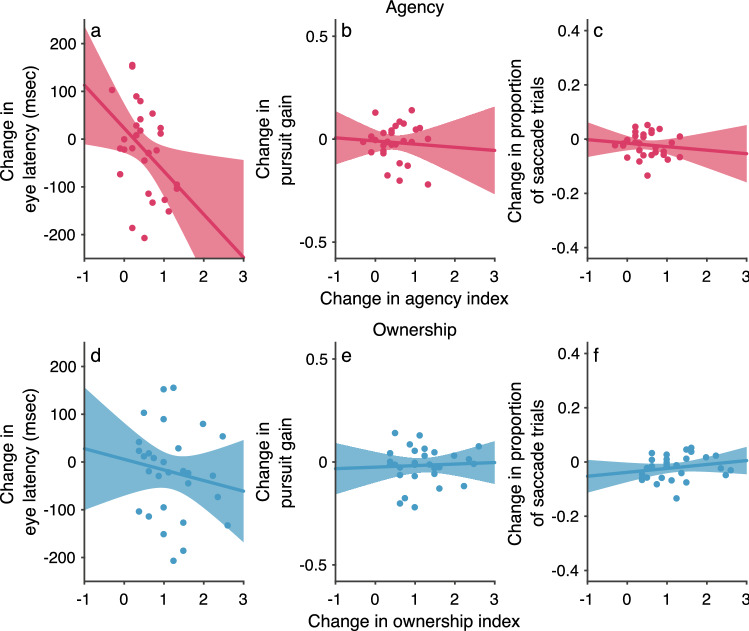
Correlation between body awareness and eye–hand coordination performance in experiment 2 (*n* = 29). (**a**–**c**) Agency. (**d**–**f**) Ownership. Change in eye–hand coordination performance [i.e., eye latency (**a**, **d**), pursuit gain (**b**, **e**), and proportion of saccadic trials (**c**, **f**)] was defined as the change in performance when the sense of agency or the sense of body ownership became stronger in the active-synchronous condition compared with the active-asynchronous condition.

Furthermore, I analyzed the correlation between the results for strength of sense of agency and eye–hand coordination performance in experiment 2 in the same manner as that in experiment 1. I again found that eye latency was significantly correlated with sense of agency (Fig. 5a: Spearman correlation test, *r*_*s*_ = -0.40, *N* = 29, *P* < 0.05), but pursuit gain and the proportion of saccadic trials were not (Fig. 5b,c: *r*_*s*_ = 0.10, *N* = 29, *P* = 0.61 *n.s.* for pursuit gain and *r*_*s*_ = -0.13, *N* = 29, *P* = 0.50 *n.s.* for proportion of saccadic trials). There were no significant correlations between sense of ownership and eye–hand coordination for eye latency (*r*_*s*_ = -0.21, *N* = 29, *P* = 0.27 *n.s.*), pursuit gain (*r*_*s*_ = 0.033, *N* = 29, *P* = 0.86 *n.s.*), and the proportion of saccadic trials (*r*_*s*_ = 0.29, *N* = 29, *P* = 0.13 *n.s.*) (Fig. 5d–f). Moreover, the ownership rating was higher in experiment 2 than in experiment 1. For example, the median of the ownership rating in the active-synchronous condition was around + 1 in experiment 2 (Fig. 4a), but it was below + 1 in experiment 1 (Fig. 2a). Despite of the difference in ownership rating between experiments 1 and 2, the correlations between sense of ownership and eye–hand coordination performance were quite similar between these experiments (Figs. 3 and 5). This suggests that sense of ownership does not affect eye–hand coordination. Also, the difference in movement distance between the active and passive conditions was not correlated with sense of agency or with sense of body ownership (see Supplementary Fig. 8 for details). Therefore, these results cannot be explained by adaptation to the position of the hand that results from the RHI procedure.

## Discussion

This study investigated how awareness of one’s body parts influences motor control. In everyday manual tasks, we have senses of agency and body ownership for our visible hands. The occurrence of sense of agency has been explained by using the comparator model^3,53^. It is generally thought that the comparator model of agency can apply to various aspects of motor control including movement initiation and ongoing movement. Surprisingly, however, the present data on the correlation between sense of agency and eye–hand coordination revealed that the eye latency significantly correlated with the strength of sense of agency, but the pursuit gain and number of saccades did not. Eye latency is an indicator of how fast the onset of hand movements evokes pursuit eye movements in the initiation of eye–hand coordination. Eye latency can be shortened when the onset of hand movements is predicted before moving the eyes. In contrast, pursuit gain and number of saccades are indicators of how precisely pursuit eye movements track hand movements during ongoing eye–hand coordination. Thus, the present findings indicate that sense of agency improves movement initiation rather than ongoing movement, suggesting that sense of agency is only linked to predicting initiation of one’s own action. This points to a revision to the current comparator model of agency.

Previous studies have not reached consensus on whether body ownership affects the motor system. The present study may explain the discrepancies among studies. Recent behavioral studies have suggested that body ownership affects the motor system. For example, body ownership affected reaching^15,16^ and grasping^14,17^ movements and attenuating self-generated tactile sensations^18–21^. Patients with spinal cord injury^26^ or hemiplegia^27^ had a distorted sense of body ownership, implying the influence of body ownership on motor control. Neuropsychological studies have suggested that a monothematic delusion of body ownership affects motor control^22^.

However, evidence from these previous studies is inconsistent with that from other brain damage and behavioral studies, which support the opposite view that body ownership does not affect the motor system. Patients with motor paralysis experienced illusory ownership of the rubber hand^24^. Further, even in healthy participants, adjustment in sensory-motor conflict can occur in the absence of alteration of sense of body ownership^28^. When healthy participants moved a cursor on a screen using a joystick, motor control and estimation of the body’s state did not require sense of body ownership to be accurate^29,30^. Furthermore, even though participants did not move their hand in the conventional situation of measuring the RHI, a previous study identified a sense of agency as a subcomponent of embodiment^47^. However, this previous study also found that the ownership subcomponent was significantly related to proprioceptive drift, but the agency subcomponent was not. This suggests a dissociation between ownership and agency.

The discrepancies among studies may be explained by the fact that the previous studies did not separate body ownership and agency experimentally. These studies used manual behavioral tasks that involved voluntary hand movements, which generated not only a sense of body ownership but also a sense of agency. The present study addressed this issue by manipulating senses of body ownership and agency experimentally (i.e., the moving RHI), and provides evidence that body ownership alone does not affect the motor system, but agency alone does. Therefore, the studies suggesting the effects of body ownership on the motor system might be explained by sense of agency, not sense of body ownership. This may explain the discrepancies among studies.

Furthermore, the present findings suggest that there might be two sources of the body’s state to the motor system: motor commands and agency. In the present study, both agency and motor commands worked in the synchronous condition, while only agency seemed to work in the asynchronous condition. Indeed, the present results showed that participants experienced a sense of agency over the CG hand in both the active-synchronous and active-asynchronous conditions. In contrast, motor commands matched the visual feedback of the moving hand (i.e., the moving CG hand) in the active-synchronous condition, but not in the active-asynchronous condition. This mismatch in the active-asynchronous condition would prevent the use of motor commands from predicting the visual consequences of the moving CG hand^54,55^. Therefore, motor commands were not useful to generate efficient eye–hand coordination in the active-asynchronous condition. Nevertheless, efficient eye–hand coordination was observed in the active-asynchronous condition, and the eye–hand coordination performance in the active-asynchronous condition was comparable to that in the active-synchronous condition. This suggests that agency, but not motor commands, may play a critical role in generating efficient eye–hand coordination at least in the active-asynchronous condition.

These results suggest that not only motor commands but also sense of agency may be used to predict the visual consequences of the moving hand. Earlier studies suggest that the body’s state is estimated using prediction based on motor commands from voluntary actions^1,2^. The present study showed that efficient eye–hand coordination occurred when the CG hand was voluntarily moved in synchrony with the participant’s hidden hand. This suggests that motor commands from voluntary hand movements may become the dominant contributor in generating efficient eye–hand coordination, which is consistent with the results of earlier studies. If the body’s state is estimated based only on motor commands, one would expect that efficient eye–hand coordination does not occur when the tracked CG hand is voluntarily moved in asynchrony with the participant’s hidden hand because of the temporal inconsistency between the motor commands and the tracked hand. However, efficient eye–hand coordination also occurred even when the CG hand was voluntarily moved in asynchrony with the participant’s hidden hand, inducing a sense of agency for the CG hand. Thus, the present study suggests that one source of the body’s state is based on motor commands from voluntary hand movements and the other is based on sense of agency in the initiation of voluntary action.

What might be the mechanisms underlying the link between sense of agency and the motor system? When we actively move our hands, our brain predicts the consequences of our hand’s motor commands^56^. This process requires a system that captures the causal relationship between our motor commands and their consequences. Such a system is termed an internal forward model^54,57,58^. This model is embedded in the comparator model^3^. The internal forward model can be divided into two stages: the forward dynamic model and forward sensory model^1^. The forward dynamic model predicts the future position of the hand based on a copy of the motor command (efference copy)^59,60^, and the forward sensory model predicts sensory feedback based on this efference copy. Previous studies have demonstrated that the eye movement system uses information from hand motor commands to coordinate eye and hand movements in finger tracking^37–39^. This ability to coordinate eye and hand movements is thought to be achieved using the forward dynamic model^61^. To generate anticipatory smooth pursuit eye movements for stabilizing the image of the tracking hand on the fovea, the forward dynamic model predicts the future position of the voluntarily moved hand. However, the present study showed that anticipatory smooth pursuit is initiated more rapidly when a sense of agency is experienced for the hand. This suggests that sense of agency exerts its influence on the forward dynamic model to predict the future position of the hand in the initiation of eye movements. Thus, the present study extends the functional significances of agency in internal forward models.

How can sense of agency affect the forward dynamic model from a computational perspective? The core of sense of agency is the association between a voluntary action and an outcome. Sense of agency is recently thought to be produced from a subjective consequence of an association between action and outcome rather than from a physical temporal match between predicted and actual outcome^3^. Early research shows that sense of agency can be quantified as a perceived compression of the time interval between action and outcome, even though a sensory feedback is physically delayed compared with the onset of voluntary action^62^. Thus, the most likely explanation is that voluntary hand movement may compress the subjective time interval between the initiation of the hand movement and the perceived onset of subsequent CG hand movements in the present study, favoring a timing that is shifted toward the onset of the CG hand movement instead of the real hand movement. The forward dynamic model may use this estimate to generate sensory predictions when new motor commands are generated. This proposal is consistent with the present finding that the stronger the subjective experience of agency, the shorter the eye latency.

The present findings suggest a prospective view of explicit sense of agency. Sense of agency has been generally explained by the comparator model^3^. According to this model, sense of agency is assumed to be generated in all aspects of motor control including movement initiation and ongoing movement. The model demands a physical temporal match between predicted and actual outcome to generate sense of agency. However, the present study indicates that sense of agency improves movement initiation rather than ongoing movement. This is demonstrated by the finding that eye latency rather than pursuit gain and number of saccades correlates with the strength of sense of agency. Since eye latency becomes shortened when the onset of hand movements is predicted before moving the eyes, this finding suggests that sense of agency is linked to the predictive, but not physical, outcomes of actions, and points to a revision to current understanding of the comparator model. Moreover, given that the present study measured the subjective rating of sense of agency through the questionnaire items, these findings suggest that explicit sense of agency is linked to the predictive outcomes of actions. Thus, the present study supports the emerging view that sense of agency depends on prospective signals^10,13^, and suggests that the prospective theory of sense of agency can be applied to not only implicit but also explicit sense of agency.

The neural bases of eye–hand coordination have been examined in monkeys and humans. The evidence consistently suggests that the cerebellum is likely part of a control system of coordinated eye–hand movements^61,63,64^. Moreover, the cerebellum contains forward models of the motor system^65^. However, it has been suggested that the internal spatial state of the body is represented in the parietal cortex^66^. The parietal region plays a key role in comparing signals relevant to sense of agency^3^. Interestingly, activations in the parietal cortex strongly depend on the results of the comparison between intentional signals and sensory feedback signals, rather than on the process of comparison itself^3^. This may explain why the sense of agency was experienced even when the CG hand was voluntarily moved, but asynchronously with the participant’s hidden hand. The parietal cortex is anatomically connected to the frontal and prefrontal motor areas. The frontal and prefrontal motor areas play a crucial role in planning and initiating a voluntary action^67^. Information from the parietal areas underlying sense of agency could reach the cerebellum through these neural structures^68,69^. Thus, these findings suggest that the representation of the body associated with the subjective experience of initiating a voluntary action may act as a source of the body’s state to the forward models.

The relationship between sense of agency and sense of body ownership has been described by three different models^70–72^. According to an additive model, agency and body ownership are strongly related, and the ability to control actions is a cue to body ownership^70^. According to an independence model, agency and body ownership are qualitatively different experiences, underpinned by distinct brain systems^71^. According to an interactive model, agency and body ownership represent different experiences with specific and exclusive brain areas but they partly overlap at the neural level^72^. The present study shows the relationship between agency and eye–hand coordination, but no relationship between body ownership and eye–hand coordination. These findings suggest that the ability to coordinate eye with hand movements is not a cue to body ownership, supporting the independence model. However, the present study also shows that the relationship between agency and eye–hand coordination is revealed only for movement initiation. This implies that the application of the independence model to body awareness might be limited to a specific aspect of motor control such as initiation of voluntary actions.

Sense of agency is important in rehabilitation medicine and sports science. To overcome motor dysfunctions, effective rehabilitation techniques need to be developed for patients who cannot move their body parts voluntarily. No effective techniques have yet been developed in rehabilitation medicine^73^. Recent research in sports science is also exploring ways to improve the performance of athletes based on their reading of their body in their mind^74^. The present results reveal that a sense of agency over an artificial hand improves the initiation of a motor action. Although this study did not address the relationship with rehabilitation medicine and sports science, I suggest that the approach of artificially inducing agency over a paralyzed body part, or a healthy body part that does not move ideally in sports, might be useful for improving the performance of patients with motor paralysis and athletes. Future research is needed to examine the direct relationship with rehabilitation medicine and sports science.

## Methods

### Participants

Twenty participants (7 women, 13 men; mean age 22.75 [range 19–28] years) were recruited in experiment 1, and 29 participants (13 women, 16 men; mean age 22.66 [range 19–28] years) were recruited in experiment 2. All participants had normal or corrected-to-normal vision, and gave informed consent in accordance with the Code of Ethics of the World Medical Association (Declaration of Helsinki). The study was approved by the Ethics Committee of the Graduate School of Information Sciences, Tohoku University. All methods were carried out in accordance with relevant guidelines and regulations.

### Apparatus and stimuli

Participants placed their right hand on a table and wore a head-mounted display (HMD; SMI Eye Tracking HMD based on the HTC Vive, comprising dual 3.6-inch microdisplays with 1080 × 1200 pixels per eye and 110° diagonal field-of-view; SensoMotoric Instruments, Teltow, Germany) that displayed visual stimuli in stereoscopic three-dimensions. The HMD was equipped with an eye tracker that measured the gaze position of the participant’s eyes at a sampling rate of 250 Hz. The HMD was covered with black tissue to occlude all surrounding visual input and was equipped with a customized forehead rest. The arm of a PHANToM force-feedback device (3D Systems, Cary, NC, USA) was attached to the participant’s right index finger. The force-feedback device was used to measure the position of the participant’s right hand at a sampling rate of 90 Hz. Participants viewed a realistic life-sized three-dimensional computer graphics (CG) hand placed on a gray surface through the HMD (Fig. 1). The gray surface was spatially aligned with the real table, where participants placed their hand. A white starting point and a turning point were presented on the gray surface.

To manipulate sense of ownership, I took advantage of the fact that sense of ownership is elicited with the CG hand movement and the CG hand is aligned with the participant’s hand^36^. To manipulate sense of agency, I took advantage of the fact that sense of agency is elicited when the CG hand is actively moved^36,40^. Four conditions were defined. I systematically manipulated the mode of the participant’s hand movement (active or passive hand movements) and the relative timing between the movements of the participant’s hand and the CG hand (synchronous or asynchronous): (i) active-synchronous—the participant actively moved their own hand, with which the CG hand moved in synchrony; (ii) active-asynchronous—the participant actively moved their own hand but the CG hand movements were temporally delayed by 0.5 s; (iii) passive-synchronous—the participant’s hand was passively moved by the arm of the force-feedback device, with which the CG hand moved in synchrony; (iv) passive-asynchronous—the participant’s hand was passively moved by the arm of the force-feedback device, and the CG hand movements were temporally delayed by 0.5 s. In the passive movement conditions, the participant’s whole right arm was moved by the force-feedback device, during which the participants were instructed to relax their hand and muscles and to not react against the movements of the device arm.

### Procedures

A session consisted of a hand-movement task and a finger-tracking task. Each session started with an eye-movement calibration procedure where the participant fixated on ten dots presented sequentially on horizontal and vertical center lines of the display and pushed a button upon completion of each fixation. The CG hand was presented 10° to the left of the participant’s unseen right hand. For each trial, participants performed the hand-movement task before the finger-tracking task. In the hand-movement task, the participant’s right hand repeatedly moved between the starting point and the turning point for 60 s (10 s after the first trial), either synchronously or asynchronously with the movements of the CG hand, while listening to a sound cue at 0.4 Hz (In experiment 1, the right hand moved to either the left or right. In experiment 2, the right hand moved to either the back or front). The use of the sound cue helped participants maintain the same speed of their own side-to-side hand movements in the active-synchronous and active-asynchronous conditions. The synchronous movements of the CG hand served to induce the body ownership illusion, as shown by previous studies^36,40^, with either active or passive movements of the participant’s hand. The actively moved CG hand served to induce a sense of agency over the CG hand with either synchronous or asynchronous movements of the participant’s hand, as shown by a previous study^40^. Upon completion of the hand-movement task, participants initiated a finger-tracking task trial by pressing a button using their left hand. After a 400-ms delay, a sound cue indicated the start of the trial. The participant’s right hand then moved to the left or right from the starting point to the turning point and back to the starting point. The CG hand moved synchronously or asynchronously with the participant’s unseen right hand for different sessions of trials. The direction of motion was constant in a given session of trials and alternated between sessions. Participants were instructed to make a smooth movement; that is, turn smoothly without stopping the movement at the turning point. There were no further constraints on the speed of their movement. They were instructed to keep their gaze on the index finger of the CG hand.

After the trials, the participants answered questionnaire items to rate subjective aspects of the CG hand. The questionnaire items for ownership were similar to those used in Botvinick and Cohen’s study^75^, and the questionnaire items for agency were similar to those used in Kalckert and Ehrsson’s study^36^. In experiment 1, for both cases, the first two questionnaire items were relevant to the illusion and the remaining three served as controls. In experiment 2, for both ownership and agency, the first four questionnaire items were relevant to the illusion and the remaining four served as controls. The order of conditions was counterbalanced across participants. Each condition consisted of 40 trials (20 repetitions of each hand-moving direction). Details of the questionnaire items are shown in Tables 1 and 2. The order of the questionnaire items was counterbalanced across participants.

### Calculation of ownership and agency indexes

The indexes were calculated by subtracting the median of the score of the control questions from the median of the score of the illusion questions. Participants’ responses to the control questions eliminate any effects of suggestibility or compliance.

### Analysis of eye and hand movements

Movements of the participant’s right index finger were tracked at 90 Hz with a PHANToM Premium 1.5 force-feedback device (3D Systems, Cary, NC). The arm of the force-feedback device was attached to the tip of the participant’s right index finger. Eye movements were recorded at 250 Hz with an SMI Eye Tracking HMD based on the HTC Vive (SensoMotoric Instruments, Teltow, Germany). The HMD was equipped with a customized forehead rest to limit head movements. The distance between the eyes and the CG hand was 40 cm. The eye position and finger position traces were checked for all trials. Trials in which participants failed to make the correct movements of the eye or finger were rejected (10.1%). The regression-based method was used, as described in Schutz et al.^76^, to analyze the eye and finger movement onset in individual trials. Velocity signals were calculated by differentiating the position data and were low-pass filtered below 10 Hz. Regression lines were fitted with 80-ms length to the velocity trace and were discarded with *R*^2^ < 0.7 or with a slope below 10°/s^2^. The regression line with the highest *R*^2^ value was selected. The time corresponding to zero velocity was defined as the movement onset. Saccades were detected by using a velocity threshold of 30°/s.

### Eye and hand data preprocessing

For the finger-tracking task, the eye and finger positions were recorded. Based on these position data, I measured eye latency (eye movement onset time relative to CG hand movement onset time), pursuit gain (eye velocity divided by CG hand velocity after excluding saccades in velocity traces), and the proportion of saccadic trials (the number of trials that had at least one saccade within a 100-ms time window was counted and then divided by the total number of trials). Eye latency, pursuit gain, or the proportion of saccadic trials was excluded from analysis when these data were not within 3 standard deviations from average. According to this criterion, one participant was excluded from Spearman’s rank correlation analysis for both eye latency and pursuit gain as well as the questionnaire response in experiment 1 (Fig. 3a,b,d,e).

### Statistical tests

To determine whether ownership index and agency index differed among four conditions (active-synchronous, active-asynchronous, passive-synchronous, and passive-asynchronous), Friedman tests were performed with conditions as factors. The standard score (z-score) was calculated to compare the agency index to the ownership index, and then the difference in the standardized index was defined as the standardized ownership index subtracted from the standardized agency index. To analyze these indexes, median tests were performed with the Bonferroni correction for multiple comparisons. For eye-tracking data, I analyzed whether standard deviation was statistically different across conditions. This analysis showed that there were significant differences in standard deviation for pursuit gain and saccadic corrections, but not eye latency, across conditions (Hartley test, *F*_*max*_(4,19) = 4.39, *P* < 0.05 for pursuit gain; *F*_*max*_(4,19) = 10.93, *P* < 0.05 for saccadic corrections in experiment 1). Therefore, I used a non-parametric test for statistical analysis of eye-tracking data. To analyze the eye-tracking data, the Friedman test was performed with conditions as factors. Post hoc comparisons were performed using Friedman tests with the Bonferroni correction for multiple comparisons. For the ownership and agency indexes and the eye-tracking data, multiple regression analyses were performed to estimate interaction effects between factors^77^. Linear regression analyses were performed between the participants’ agency or ownership index and their eye–hand coordination performance. Regression coefficients were tested against zero with two-tailed Spearman correlation tests. The data were processed in MATLAB R2019a and analyzed using SPSS (Version 25, IBM).

## Supplementary information

Supplementary Information 1.

Supplementary Information 2.

Supplementary Information 3.

## Data Availability

The data that support the findings of this study are available from the corresponding author upon reasonable request.
