# Supplementary material for: Awareness of voluntary action, rather than body ownership, improves motor control
Source: Sci Rep. 2021 Jan 11;11:418. doi: 10.1038/s41598-020-79910-x (PMC7801649; doi:10.1038/s41598-020-79910-x)
Supplement: Supplementary file 3 — Supplementary Information 3. [file 41598_2020_79910_MOESM3_ESM.pdf]

Supplementary Information

Awareness of voluntary action, rather than body ownership, improves motor control

Kazumichi Matsumiya

Email: matsumiya@tohoku.ac.jp

This PDF file includes:

Supplementary Results
Supplementary Figures 1 to 8
Legends for Supplementary Movies 1 and 2

Supplementary Results

Results of experiment 2 (Fig. 4). Fig. 4a and b shows the standardized strength of the rated ownership and agency of the CG hand as box-and-whisker plots for the four conditions. In Fig. 4a, the standard score (z-score) was calculated for the ownership and agency indexes each, which was defined as the standardized index. The horizontal black bars represent the medians, and the boxes denote the interquartile ranges and extend from the first to the third quartile. The whiskers extend to the farthest data points within 1.5 times the interquartile range from the top and bottom edges of the boxes. The individual open circle represents an outlier. In Fig. 4b, the standardized ownership index was subtracted from the standardized agency index, which was used as a difference in standardized index. The colored bars in Fig. 4b represent the medians of the difference in standardized index, and the error bars represent the interquartile ranges and extend from the first to the third quartile.

For ownership rating, analysis of the questionnaire data revealed a significant main effect of timing condition (Fig. 4a, blue; synchronous and asynchronous; Friedman test, $\chi^2 = 23.23$, $df = 1$, $n = 29$, $P < 0.001$), but not of action condition (active and passive; $\chi^2 = 3.38$, $df = 1$, $n = 29$, $P = 0.066$ *n.s.*). There was no significant interaction between action and timing conditions (Regression analysis, $t_{28} = 1.80$, $P = 0.074$ *n.s.*). For agency rating, the analysis revealed a significant main effect of action condition (Fig. 4a, pink; active and passive; $\chi^2 = 67.17$, $df = 1$, $n = 29$, $P < 0.001$), but not of timing condition (synchronous and asynchronous; $\chi^2 = 3.08$, $df = 1$, $n = 29$, $P = 0.079$ n.s.). There was no significant interaction between action and timing conditions ($t_{28} = 1.84$, $P = 0.068$ *n.s.*). The difference in standardized indexes significantly differed from zero (median test: $z = 29.35$, $n = 29$, $P < 0.001$), and was significantly larger in the active-asynchronous condition than in the passive-synchronous condition (Bonferroni corrected median test: $z = 27.62$, $n = 29$, $P < 0.001$) (Fig. 4b).

In Fig. 4d, analysis of eye latency revealed a significant main effect of mode (active vs passive) condition (Fig. 4b; Friedman test, $\chi^2 = 63.03$, $df = 1$, $n = 29$, $P < 0.001$), but no significant main effect of timing (synchronous vs asynchronous) condition ($\chi^2 = 0.05$, $df = 1$, $n = 29$, $P = 0.83$ *n.s.*). There was no significant interaction between action and timing conditions (Regression analysis, $t_{28} = -1.45$, $P = 0.15$ *n.s.*). Eye latency was significantly longer in the passive-synchronous condition than in the active-synchronous and active-asynchronous conditions (Bonferroni corrected Friedman test: $z = -5.70$, $P < 0.001$ and $z = -5.29$, $P < 0.001$, respectively). Eye latency in the passive-synchronous condition was similar to that in the passive-asynchronous condition. Furthermore, eye latency in the active-asynchronous condition was almost the same as that in the active-synchronous condition but was significantly shorter than that in the passive-asynchronous condition ($z = -5.09$, $P < 0.001$).

In Fig. 4f, analysis of pursuit gain revealed a significant main effect of mode condition ($\chi^2 = 84.92$, $df = 1$, $n = 29$, $P < 0.001$), but not of timing condition ($\chi^2 = 2.54$, $df = 1$, $n = 29$, $P = 0.11$ *n.s.*). There was no significant interaction between action and timing conditions ($t_{28} = 0.27$, $P = 0.79$ *n.s.*). Pursuit gain was not significantly different between the passive-synchronous and passive-asynchronous conditions (Bonferroni corrected Friedman test: $z = -1.73$, $P = 0.50$ *n.s.*). Pursuit gain in the passive-synchronous condition was significantly less than that in the active-synchronous ($z = 6.51$, $P < 0.001$) and active-asynchronous conditions ($z = 7.02$, $P < 0.001$), and was significantly different between the active-asynchronous and passive-asynchronous conditions ($z = 5.29$, $P < 0.001$). Pursuit gain in the active-asynchronous condition was comparable to that in the active-synchronous condition. There was a significant difference between the active-synchronous and passive-asynchronous conditions ($z = 4.78$, $P < 0.001$).

In Fig. 4h, analysis of the proportion of saccadic trials revealed a significant main effect of mode condition (Friedman test: $\chi^2 = 85.53$, $df = 1$, $n = 29$, $P < 0.001$), but not of timing condition ($\chi^2 = 0.83$, $df = 1$, $n = 29$, $P = 0.36$ *n.s.*). There was no significant interaction between action and timing conditions ($t_{28} = -0.60$, $P = 0.55$ *n.s.*). There was no significant difference in the proportion of saccadic trials between the passive-synchronous and passive-asynchronous conditions (Bonferroni corrected Friedman test: $z = -0.31$, $P = 1.0$ *n.s.*) and between the active-synchronous and active-asynchronous conditions ($z = -0.92$, $P = 1.0$ *n.s.*). There were significant differences between the active-synchronous and passive-synchronous conditions ($z = -6.20$, $P < 0.001$), between the active-synchronous and passive-asynchronous conditions ($z = -6.51$, $P < 0.001$),

between the active-asynchronous and passive-synchronous conditions ($z = -5.29$, $P < 0.001$), and between the active-asynchronous and passive-asynchronous conditions ($z = -5.59$, $P < 0.001$).

Ownership and agency questionnaires in experiment 1 (Supplementary Fig. 1).
Supplementary Figs. 1a and d show the questionnaire responses for the illusion and control items in the four conditions for ownership and agency questionnaires. Supplementary Fig. 1b shows the ownership index in the active-asynchronous condition plotted against the ownership index in the active-synchronous condition for individual participants. Supplementary Fig. 1c shows the ownership index in the passive-asynchronous condition plotted against the ownership index in the passive-synchronous condition for individual participants. Supplementary Fig. 1e shows the agency index in the passive-synchronous condition plotted against the agency index in the active-synchronous condition for individual participants. Supplementary Fig. 1f shows the agency index in the passive-asynchronous condition plotted against the agency index in the active-asynchronous condition for individual participants. The square symbol in Supplementary Figs. 1b–f represents the median.

The ownership index was significantly higher in the active-synchronous condition than in the active-asynchronous condition (Supplementary Fig. 1b: Bonferroni corrected Friedman test, $z = 3.49$, $P = 0.003$). The ownership index was significantly higher in the passive-synchronous condition than in the passive-asynchronous condition (Supplementary Fig. 1c: $z = 2.88$, $P = 0.024$). The agency index was significantly higher in the active-synchronous condition than in the passive-synchronous condition (Supplementary Fig. 1e: $z = 4.16$, $P < 0.001$). Furthermore, there was a significant difference in the agency index between the active-asynchronous and passive-asynchronous conditions (Supplementary Fig. 1f: $z = 3.06$, $P = 0.013$).

Ownership and agency questionnaires in experiment 2 (Supplementary Fig. 4).
Supplementary Figs. 4a and d show the questionnaire responses for the illusion and control items in the four conditions for ownership and agency questionnaires. Supplementary Fig. 4b shows the ownership index in the active-asynchronous condition plotted against the ownership index in the active-synchronous condition for individual participants. Supplementary Fig. 4c shows the ownership index in the passive-asynchronous condition plotted against the ownership index in the passive-synchronous condition for individual participants. Supplementary Fig. 4e shows the agency index in the passive-synchronous condition plotted against the agency index in the active-synchronous condition for individual participants. Supplementary Fig. 4f shows the agency index in the passive-asynchronous condition plotted against the agency index in the active-asynchronous condition for individual participants. The square symbol in Supplementary Figs. 4b–f represents the median.

The ownership index was significantly higher in the active-synchronous condition than in the active-asynchronous condition (Supplementary Fig. 4b: Bonferroni corrected Friedman test, $z = 5.95$, $P < 0.001$). The ownership index was significantly higher in the passive-synchronous condition than in the passive-asynchronous condition (Supplementary Fig. 4c: $z = 2.90$, $P = 0.022$). The agency index was significantly higher in the active-synchronous condition than in the passive-synchronous condition (Supplementary Fig. 4e: $z = 6.97$, $P < 0.001$). Furthermore, there was a significant difference in the agency index between the active-asynchronous and passive-asynchronous conditions (Supplementary Fig. 4f: $z = 4.53$, $P < 0.001$).

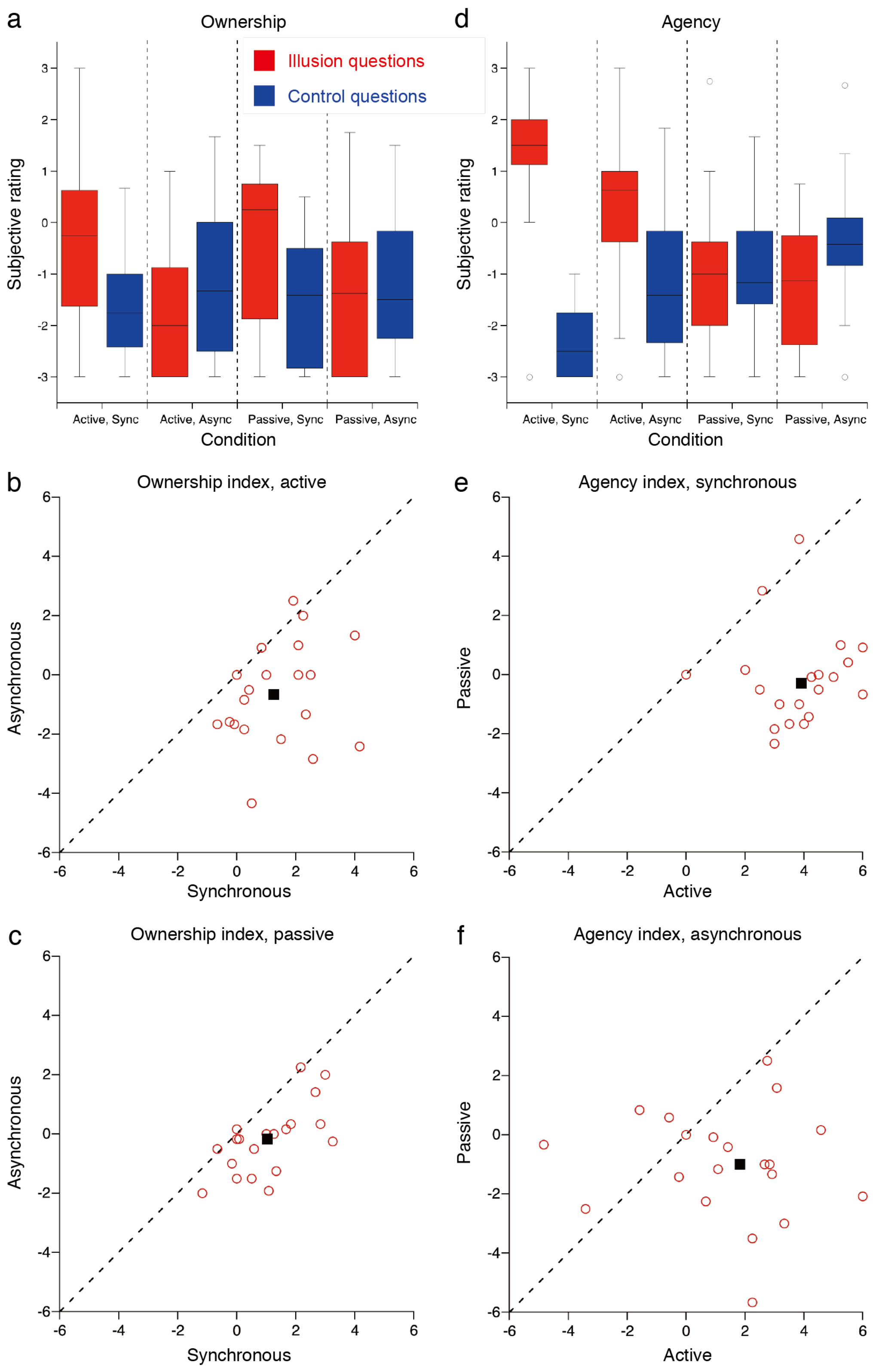

Supplementary Fig. 1. Questionnaire responses for ownership and agency in experiment 1 (*n* = 20).

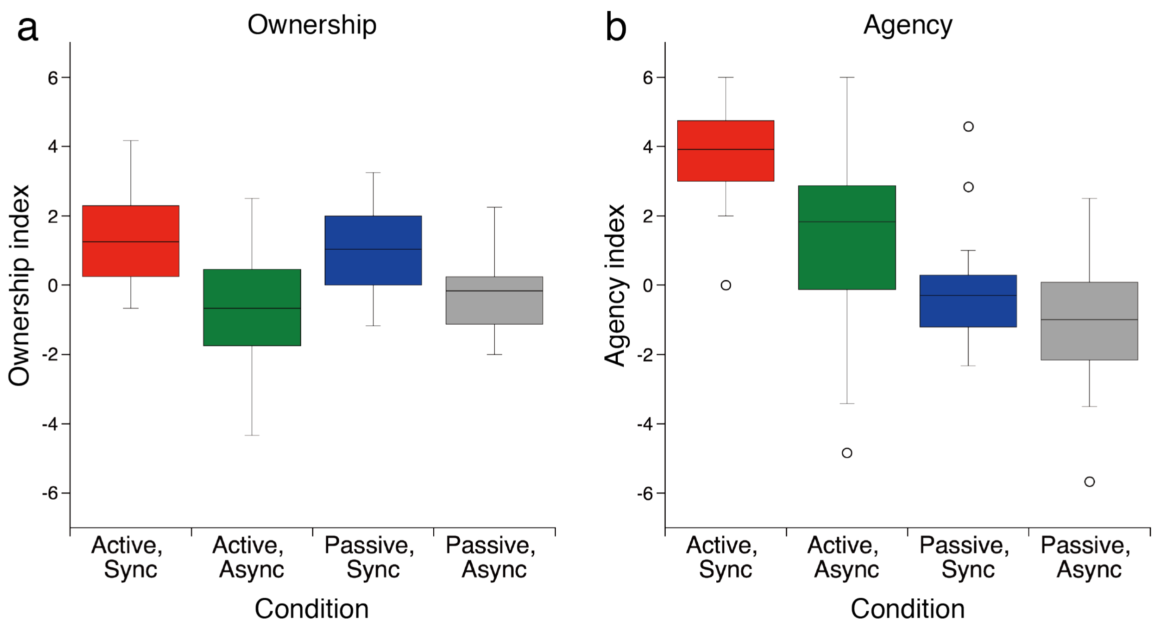

Supplementary Fig. 2. Median of ownership and agency indexes in experiment 1 (n = 20). Strength of the rated ownership and agency of the computer graphics (CG) hand is shown as box-and-whisker plots for the four conditions. Supplementary Figs. 2a and b indicate ownership index and agency index, respectively. The horizontal black bars represent the medians, and the boxes denote the interquartile ranges and extend from the first to the third quartile. The whiskers extend to the farthest data points within 1.5 times the interquartile range from the top and bottom edges of the boxes. The individual open circle represents an outlier.

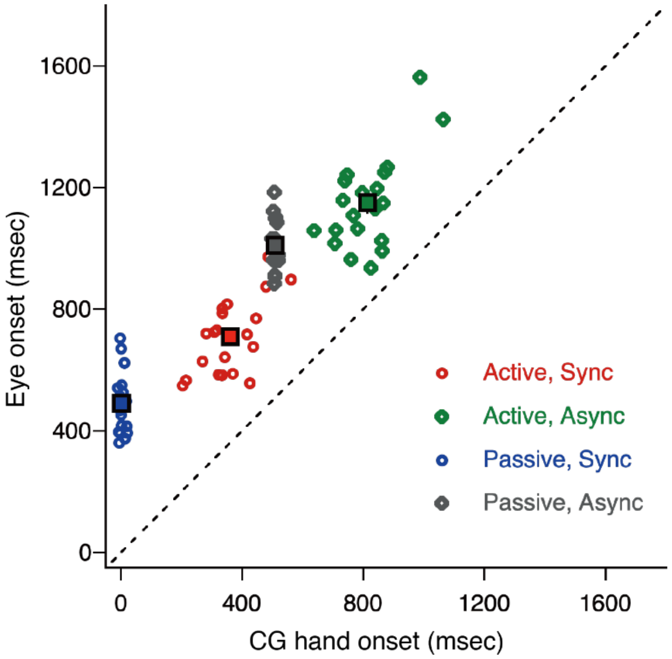

Supplementary Fig. 3. Eye onset time plotted against computer graphics (CG) hand onset time in the active-synchronous (red), active-asynchronous (green), passive-synchronous (blue), and passive-asynchronous (gray) conditions in experiment 1 (n = 20). The CG hand onset was earlier than the eye onset in all conditions. Each open symbol represents a different participant in the same color. Each square represents the average value for each color. The red, green, blue, and gray symbols represent the active-synchronous, active-asynchronous, passive-synchronous, and passive-asynchronous conditions, respectively. The squares represent the mean $\pm$ standard error.

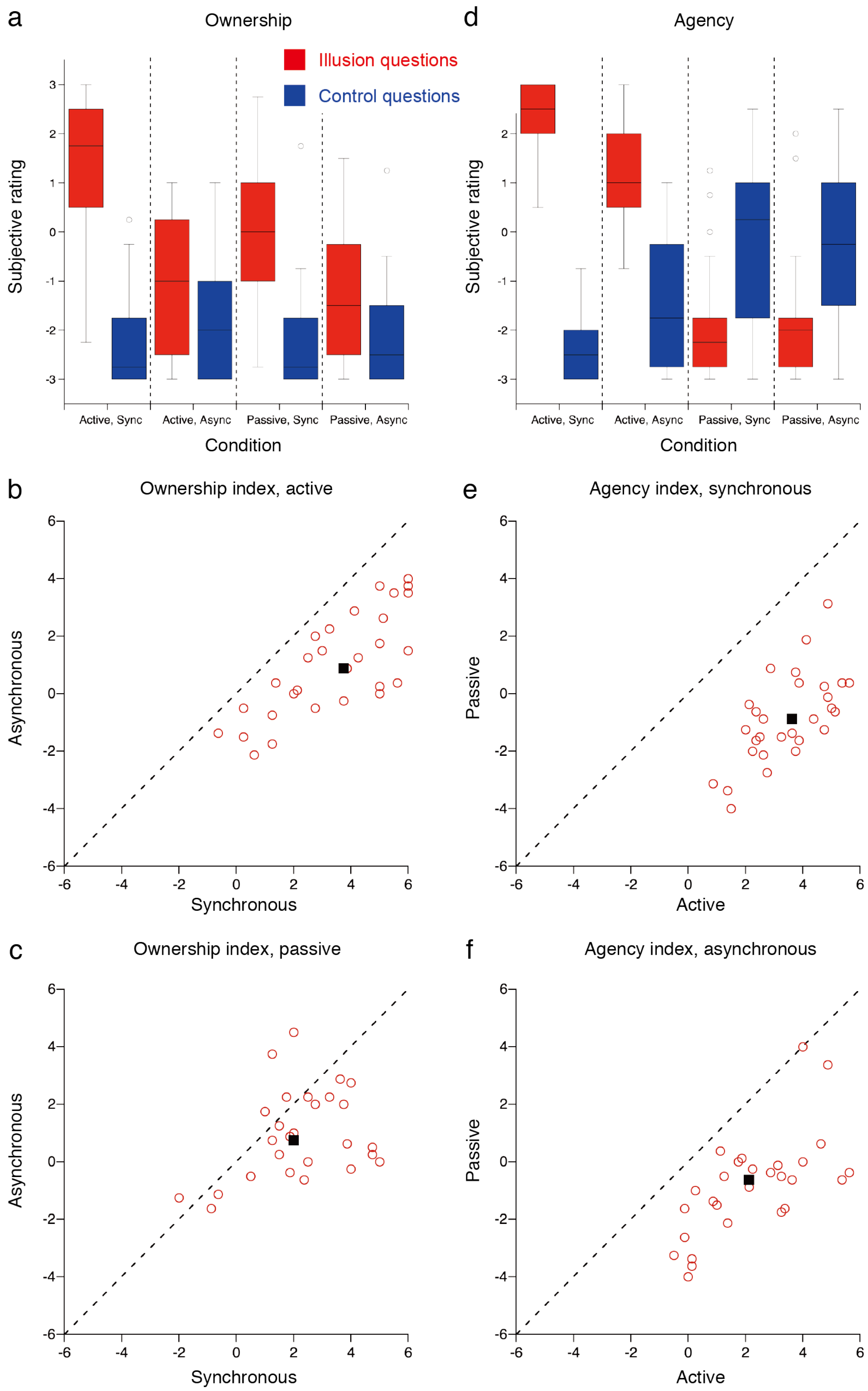

Supplementary Fig. 4. Questionnaire responses for ownership and agency in experiment 2 (n = 29).

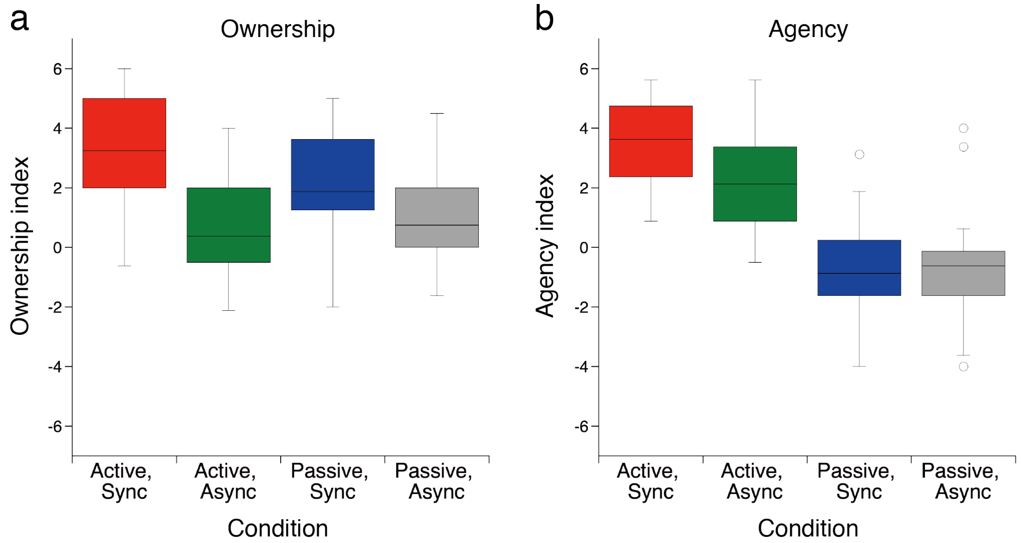

Supplementary Fig. 5. Median of ownership and agency indexes in experiment 2 (n = 29). Strength of the rated ownership and agency of the computer graphics (CG) hand is shown as box-and-whisker plots for the four conditions. Supplementary Figs. 5a and b indicate ownership index and agency index, respectively. The horizontal black bars represent the medians, and the boxes denote the interquartile ranges and extend from the first to the third quartile. The whiskers extend to the farthest data points within 1.5 times the interquartile range from the top and bottom edges of the boxes. The individual open circle represents an outlier. The ownership index tended to be lower in experiment 1 than in experiment 2 for the active-sync condition in particular. This may be because of the individual differences, since participants differed between experiments 1 and 2.

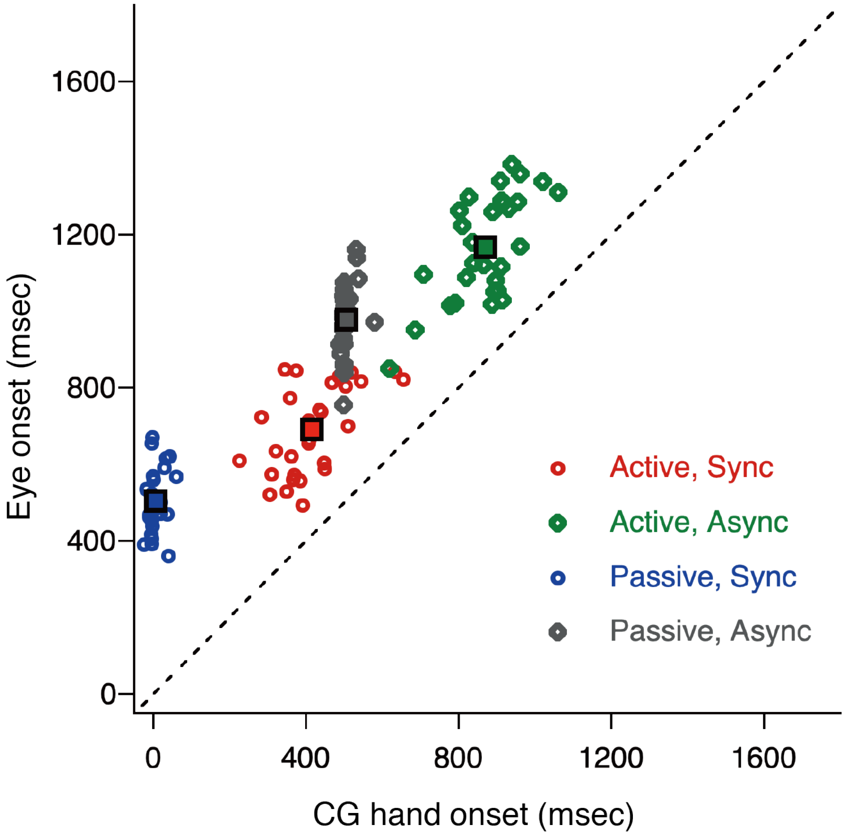

Supplementary Fig. 6. Eye onset time plotted against computer graphics (CG) hand onset time in the active-synchronous (red), active-asynchronous (green), passive-synchronous (blue), and passive-asynchronous (gray) conditions in experiment 2 (*n* = 29). The CG hand onset was earlier than the eye onset in all conditions. Each open symbol represents a different participant in the same color. Each square represents the average value for each color. The red, green, blue, and gray symbols represent the active-synchronous, active-asynchronous, passive-synchronous, and passive-asynchronous conditions, respectively. The squares represent the mean ± standard error.

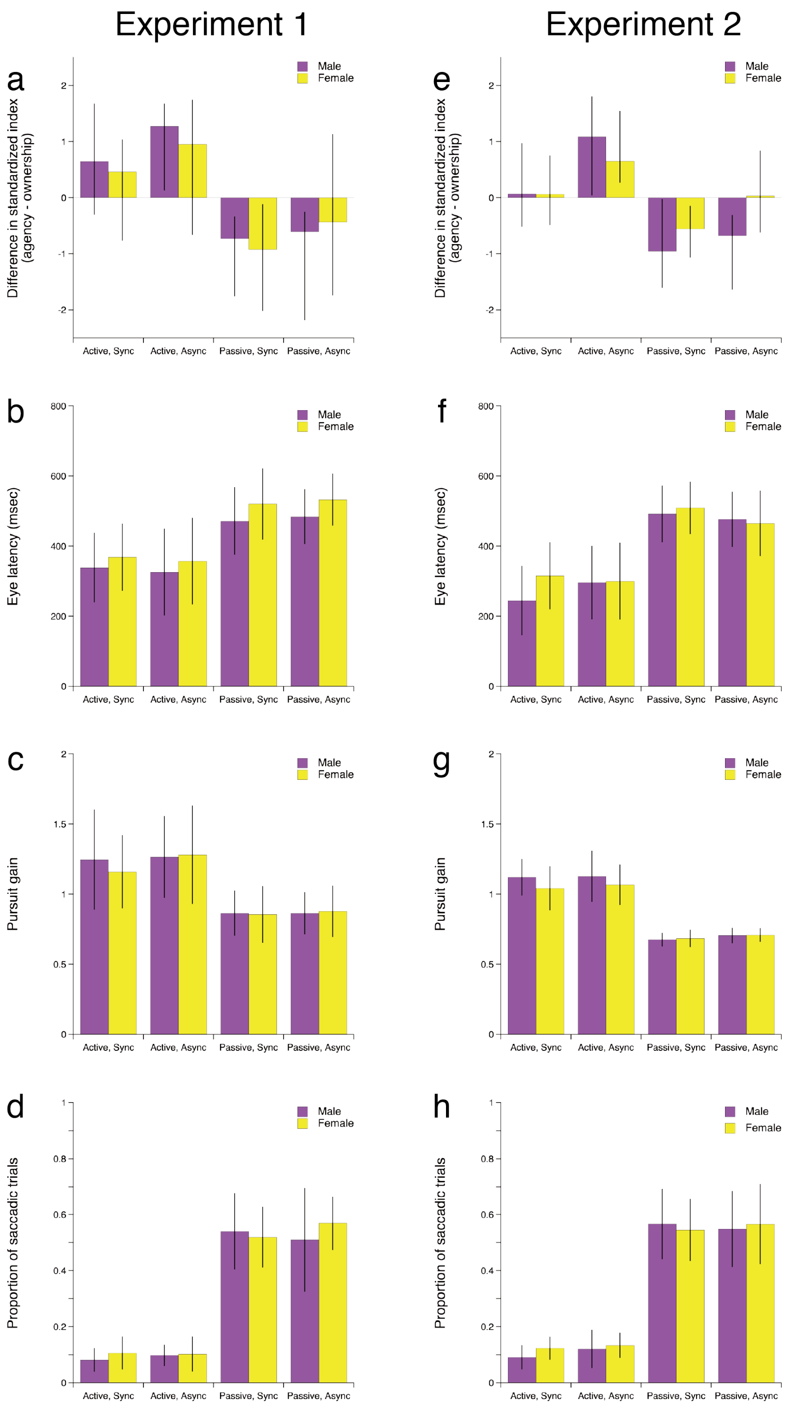

Supplementary Fig. 7. Effects of sex in experiments 1 and 2. Seven women and thirteen men participated in experiment 1, and thirteen women and sixteen men participated in experiment 2. For experiments 1 and 2, there were no significant differences in gender for questionnaire, eye latency, pursuit gain, and saccadic corrections (Kruskal-Wallis test, experiment 1: $z = 0.80$, $p = 0.37$ *n.s.* for questionnaire; $z = 1.88$, $p = 0.17$ *n.s.* for eye latency; $z = 0.041$, $p = 0.84$ *n.s.* for pursuit gain; $z = 0.23$, $p = 0.63$ *n.s.* for saccadic corrections. Experiment 2: $z = 0.94$, $p = 0.33$ *n.s.* for questionnaire; $z = 0.47$, $p = 0.49$ *n.s.* for eye latency; $z = 0.42$, $p = 0.52$ *n.s.* for pursuit gain; $z = 0.46$, $p = 0.50$ *n.s.* for saccadic corrections).

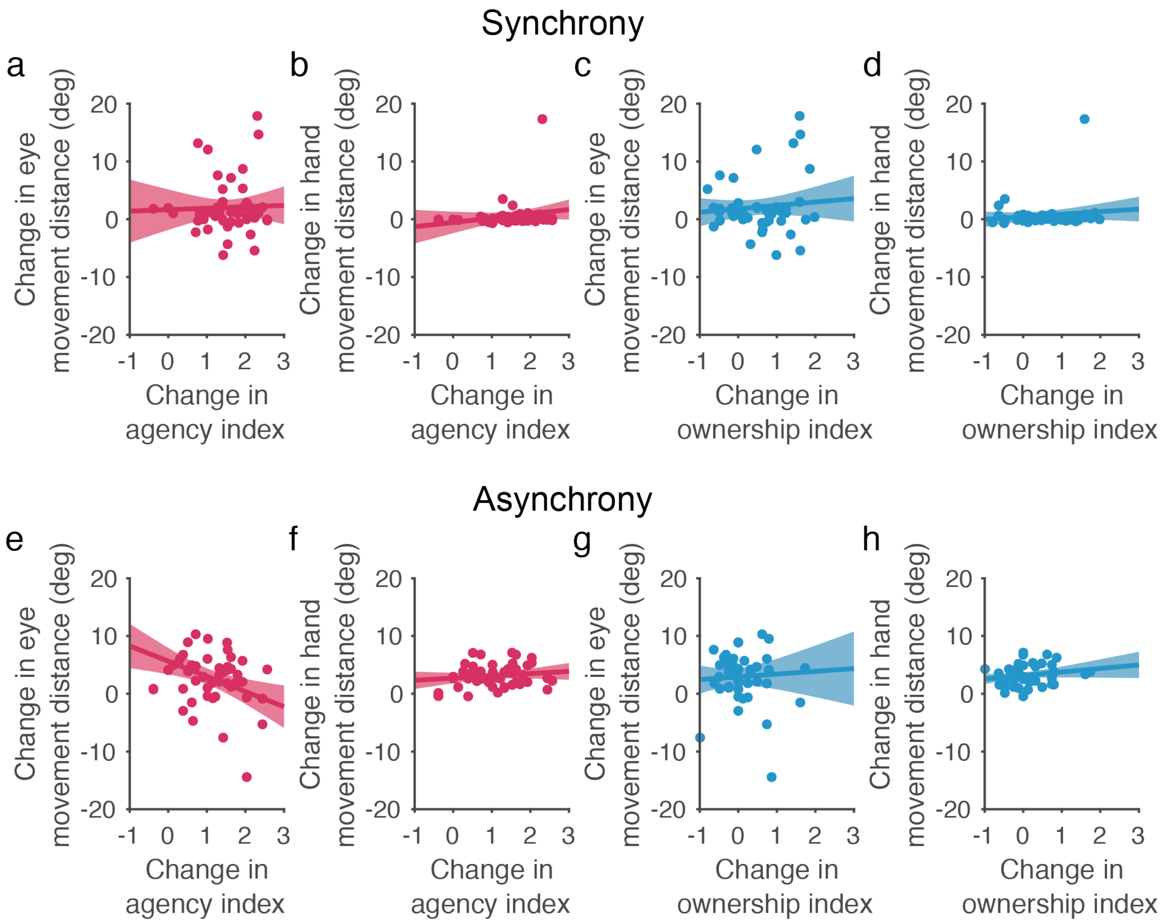

Supplementary Fig. 8. Correlation between body awareness and movement distance in synchronous (**a**–**d**) and asynchronous (**e**–**h**) conditions. The participant's right hand with their eyes moved from the starting point to the turning point and back to the starting point in each trial. I analyzed the distance from the start of movement to the turn for eye and hand movements. Change in eye or hand movement distance was defined as the change in performance when the sense of agency or the sense of body ownership became stronger in the active condition compared with the passive condition. Each graph includes the data from both experiments 1 and 2. (**a**–**d**) There were no significant correlations between sense of agency and eye movement distance (**a**; Spearman correlation test, r_s = -0.022, N = 49, P = 0.88 *n.s.*), between sense of agency and hand movement distance (**b**; r_s = 0.26, N = 49, P = 0.07 *n.s.*), between sense of ownership and eye movement distance (**c**; r_s = -0.029, N = 49, P = 0.85 *n.s.*), and between sense of ownership and hand movement distance (**d**; r_s = 0.15, N = 49, P = 0.31 *n.s.*). (**e**–**h**) There were no significant correlations between sense of agency and eye movement distance (**e**; r_s = -0.20, N = 49, P = 0.17 *n.s.*), between sense of agency and hand movement distance (**f**; r_s = 0.14, N = 49, P = 0.35 *n.s.*), between sense of ownership and eye movement distance (**g**; r_s = 0.04, N = 49, P = 0.78 *n.s.*), and between sense of ownership and hand movement distance (**h**; r_s = 0.23, N = 49, P = 0.12 *n.s.*).

Supplementary Movie 1 (separate file). Visual stimulus in experiment 1. A realistic life-sized computer graphics (CG) hand was presented through a head-mounted display. The CG hand was kept at a distance of 10° to the left of the participant's unseen right hand and placed on a gray virtual table aligned with a real table. Participants were instructed to track the tip of the right index finger of the CG hand with their eyes while their hidden right hand was moved.

Supplementary Movie 2 (separate file). Visual stimulus in experiment 2. A realistic life-sized computer graphics (CG) hand was presented through a head-mounted display. The CG hand was kept at a distance of 10° to the left of the participant's unseen right hand and placed on a gray virtual table aligned with a real table. To induce the rubber hand illusion, the participant's right hand repeatedly moved in a vertical direction for 60 s (10 s after the first trial). Then the participant's right hand moved in a horizontal direction from the starting point to the turning point and back to the starting point. Participants were instructed to track the tip of the right index finger of the CG hand with their eyes while their hidden right hand was moved.
